# *Lactobacillus rhamnosus* GG modulates innate signaling pathway and cytokine responses to rotavirus vaccine in intestinal mononuclear cells of gnotobiotic pigs transplanted with human gut microbiota

**DOI:** 10.1186/s12866-016-0727-2

**Published:** 2016-06-14

**Authors:** Haifeng Wang, Kan Gao, Ke Wen, Irving Coy Allen, Guohua Li, Wenming Zhang, Jacob Kocher, Xingdong Yang, Ernawati Giri-Rachman, Guan-Hong Li, Sherrie Clark-Deener, Lijuan Yuan

**Affiliations:** Department of Biomedical Sciences and Pathobiology, Virginia-Maryland College of Veterinary Medicine, Virginia Polytechnic Institute and State University, Integrated Life Science Building (0913), 1981 Kraft Drive, Blacksburg, VA 24061 USA; Present address: College of Animal Science and Technology, Zhejiang A & F University, Lin’an, 311300 Zhejiang Province People’s Republic of China; Department of Large Animal Clinical Sciences, Virginia-Maryland College of Veterinary Medicine, Virginia Polytechnic Institute and State University, Blacksburg, VA 24061 USA; Present address: School of Life Science and Technology, Institut Teknologi Bandung, Bandung, Indonesia

**Keywords:** *Lactobacillus rhamnosus* GG, Intestine, Gnotobiotic pigs, Inflammation, Immune response, Vaccine adjuvant

## Abstract

**Background:**

A better understanding of mechanisms underlying dose-effects of probiotics in their applications as treatments of intestinal infectious or inflammatory diseases and as vaccine adjuvant is needed. In this study, we evaluated the modulatory effects of *Lactobacillus rhamnosus* GG (LGG) on transplanted human gut microbiota (HGM) and on small intestinal immune cell signaling pathways in gnotobiotic pigs vaccinated with an oral attenuated human rotavirus (AttHRV) vaccine.

**Results:**

Neonatal HGM transplanted pigs were given two doses of AttHRV on 5 and 15 days of age and were divided into three groups: none-LGG (AttHRV), 9-doses LGG (AttHRV + LGG9X), and 14-doses LGG (AttHRV + LGG14X) (*n* = 3–4). At post-AttHRV-inoculation day 28, all pigs were euthanized and intestinal contents and ileal tissue and mononuclear cells (MNC) were collected. AttHRV + LGG14X pigs had significantly increased LGG titers in the large intestinal contents and shifted structure of the microbiota as indicated by the formation of a cluster that is separated from the cluster formed by the AttHRV and AttHRV + LGG9X pigs. The increase in LGG titers concurred with significantly increased ileal HRV-specific IFN-γ producing T cell responses to the AttHRV vaccine reported in our previous publication, suggesting pro-Th1 adjuvant effects of the LGG. Both 9- and 14-doses LGG fed pig groups had significantly higher IkBα level and p-p38/p38 ratio, while significantly lower p-ERK/ERK ratio than the AttHRV pigs, suggesting activation of regulatory signals during immune activation. However, 9-doses, but not 14-doses LGG fed pigs had enhanced IL-6, IL-10, TNF-α, TLR9 mRNA levels, and p38 MAPK and ERK expressions in ileal MNC. Increased TLR9 mRNA was in parallel with higher mRNA levels of cytokines, p-NF-kB and higher p-p38/p38 ratio in MNC of the AttHRV + LGG9X pigs.

**Conclusions:**

The relationship between modulation of gut microbiota and regulation of host immunity by different doses of probiotics is complex. LGG exerted divergent dose-dependent effects on the intestinal immune cell signaling pathway responses, with 9-doses LGG being more effective in activating the innate immunostimulating TLR9 signaling pathway than 14-doses in the HGM pigs vaccinated with AttHRV.

**Electronic supplementary material:**

The online version of this article (doi:10.1186/s12866-016-0727-2) contains supplementary material, which is available to authorized users.

## Background

Intestinal microbiota consists of approximately 10^14^ bacteria that can be classified into more than 1000 species [1]. Intestinal microbiota clearly impact mucosal immune responses in infants [2], yet our understanding of how enteric immunity is modulated by gut microbes is limited because of difficulties in performing such studies in humans, especially in infants due to ethical reasons. Germ-free pigs transplanted with human gut microbiota (HGM) provide a model system that is ideal for the study of the manifold effects of human microbiota on health and disease [3]. Human gastrointestinal tract (GI) can be colonized at birth by facultative anaerobes including *enterobacter*, *lactobacillus* and *streptococcus* in genus level, forming a reducing environment during the first week of life enabling colonization by strict anaerobes such as *bacteroides*, *clostridium*, *bifidobacterium* in genus level [4]. This microbial colonization contributes to recruitment of immune cells to the gastrointestinal tract and is a major contributor to the development of the mucosal and systemic immune systems in neonates [5]. Colonization in early infancy is crucial in relation to the final composition of the permanent microbiota in adults and also in inducing immunological maturation in the intestine and shaping future immune responses of the host [6].

Many previous studies have demonstrated that probiotic *Lactobacillus rhamnosus* GG (LGG) strain has beneficial effects on intestinal function, including stimulating development and mucosal immunity, maintaining and improving intestinal barrier function, and prolonging remission in ulcerative colitis and pouchitis [7]. Studies have also demonstrated the adjuvant effect of LGG in enhancing the immunogenicity of rotavirus, influenza virus, poliovirus, and *Salmonella typhi* Ty21a vaccines [8]. Probiotics modulate immunity in the GI tract by interacting with a range of receptors on intestinal epithelial cells (IEC), M-cells and dendritic cells [9]. Probiotics also enhance immunity beyond the GI tract through interactions with the common mucosal immune system.

Microorganisms can be sensed via pattern recognition receptors (PRRs) like Toll-like receptors (TLRs) to initiates innate immune response, in GI tract, thus affecting the development of the subsequent adaptive immune response. Due to the heavy bacterial antigen load in the lumen, the expression of PRRs is tightly regulated in IEC [10]. The TLR pathways activate several different signaling elements, including nuclear factor kB (NF-kB) and extracellular signal-regulated kinase (ERK)/c-Jun-NH2-kinase (JNK)/p38, which regulate many immunologically relevant proteins [11]. NF-kB activation is essential for eliciting protective antigen-specific immune responses after vaccination [12, 13]. Modulation of the signaling pathway will have significant impact on vaccine immunogenicity and efficacy.

In this study, we used HGM transplanted gnotobiotic (Gn) pigs to investigate how two different dosing regimens of LGG impacted the intestinal bacterial communities and modulated the immune signaling pathway responses to an oral attenuated human rotavirus (AttHRV) vaccine. The knowledge will facilitate the selection of proper dosage of probiotics in their applications as vaccine adjuvants and as treatments of intestinal infectious or inflammatory diseases.

## Results

### The LGG titers were the highest in AttHRV + LGG14X pigs and increased over time in all pigs

**Fig. 1:**
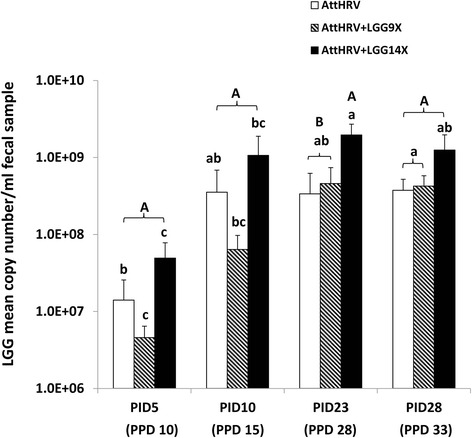
LGG fecal shedding in HGM-tranplanted Gn pigs fed none (AttHRV), 9-dose (AttHRV + LGG9X) or 14-dose (AttHRV + LGG14X) of LGG. PID, post-first-AttHRV-inoculation day. Different lowercase letters on top of bars indicate significant differences compared among time points for the same treatment group; different capital letters on top of bars indicate significant differences compared among groups at each time point, while shared letters indicate no significant difference (ANOVA-GLM, *p* < 0.05; *n* = 7–9)

The LGG titers were higher (PPD 10, 15 and 33) or significantly higher (PPD 28) in the AttHRV + LGG14X pigs than those of AttHRV and AttHRV + LGG9X pigs (Fig. 1). The LGG titers increased over time from the beginning of LGG feeding for both dosage groups. Interestingly, for the non-LGG fed AttHRV pigs, the LGG titers also increased. At PPD 33, the LGG titers were significantly higher than at PPD 10 (the first sampling time point) for all three pig groups.

### Bacterial communities in feces of HGM transplanted pigs

**Fig. 2:**
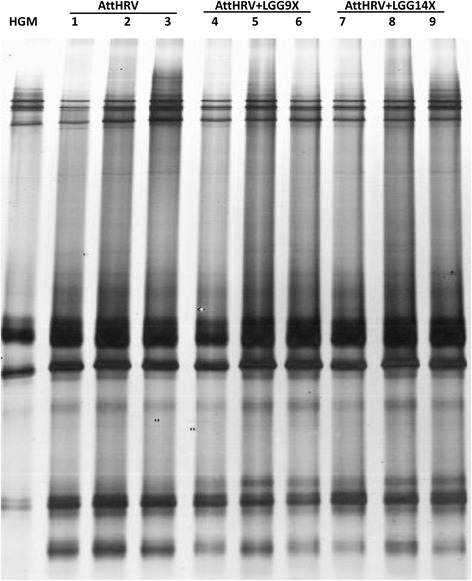
DGGE of PCR products of V6-V8 regions of 16S rDNA from bacteria in large intestinal contents of HGM-transplanted Gn pigs fed none (AttHRV), 9-dose (AttHRV + LGG9X) or 14-dose (AttHRV + LGG14X) of LGG. Pig No. 1–3 from AttHRV group; pig No. 4–6 from AttHRV + LGG9X group; and pig No. 7–9 from AttHRV + LGG14X group

**Fig. 3:**
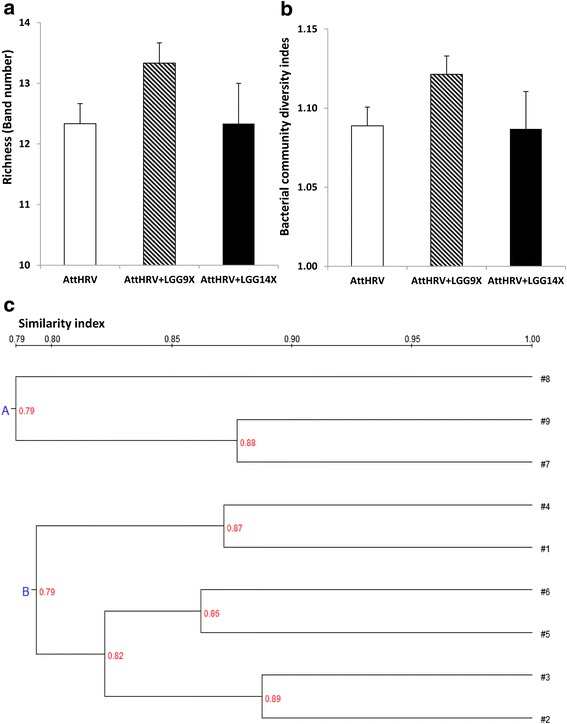
Bacterial community richness (**a**), diversity (**b**) and similarity index (**c**) of DGGE profiles in the large intestinal contents of HGM-transplanted Gn pigs fed none (AttHRV), 9-doses (AttHRV + LGG9X) or 14-doses (AttHRV + LGG14X) of LGG. Pig No. 1–3 from AttHRV group; pig No. 4–6 from AttHRV + LGG9X group; and pig No. 7–9 from AttHRV + LGG14X group

The DGGE profile of the HGM transplanted Gn pigs at PPD 33 are showed in Figs. 2 and 3. There is no significant difference in species richness (DGGE band number, Fig. 3a) and Shannon index of diversity (Fig. 3b) among different treatment groups. However, there is a trend for higher richness and diversity in the AttHRV + LGG9X pigs than the other two groups. The similarity index of the individual pigs ranged from 0.79 to 0.89.

Based on the similarity, the bacterial communities of AttHRV + LGG14X pigs (Cluster A) is separated from the bacterial communities of the AttHRV and AttHRV + LGG9X pigs (Cluster B) (Fig. 3c). Thus, 14 doses of LGG influenced the structure of the transplanted microbiota whereas 9 doses of LGG increased its richness and diversity, although not statistically significant.

### The IL-6, TNF-α, IL-10 and TLR9 mRNA levels in AttHRV + LGG9X pigs were significantly higher than the other groups

**Fig. 4:**
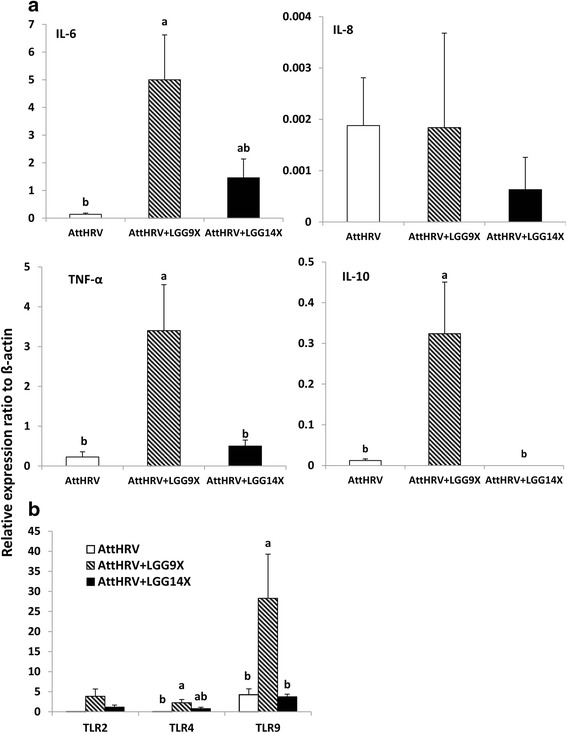
Effect of 9 (AttHRV + LGG9X) or 14 (AttHRV + LGG14X) doses of LGG on cytokine (**a**) and TLR (**b**) levels in ileal MNCs of HGM-transplanted Gn pigs. Different letters on top of bars indicate significant differences compared among groups (Kruskal–Wallis test, *p* < 0.05; *n* = 3–4), while shared letters or no letters indicate no significant difference

The relative mRNA levels of the selected cytokine in ileal MNCs were measured by real-time PCR. The IL-6 mRNA levels were significantly higher in AttHRV + LGG9X pigs than that in AttHRV pigs (Fig. 4a). No significant differences were found in IL-8 mRNA levels among different pig groups. The mRNA levels of TNF-α and IL-10 in AttHRV + LGG9X pigs were also significantly higher than those in AttHRV and AttHRV + LGG14X pigs.

The relative mRNA levels of TLR4 were slightly but statistically higher in AttHRV + LGG9X pigs than that in AttHRV pigs (Fig. 4b). The mRNA levels of TLR9 in AttHRV + LGG9X pigs were significantly higher than those in AttHRV and AttHRV + LGG14X pigs. TLR2 mRNA levels showed the same trend but did not differ significantly among the pig groups. Thus, 9 doses, but not 14 doses of LGG significantly enhanced the innate cytokine and TLR responses at transcriptional level in the AttHRV-vaccinated HGM pigs.

### The signal pathway molecule expression in the ileal MNCs

**Fig. 5:**
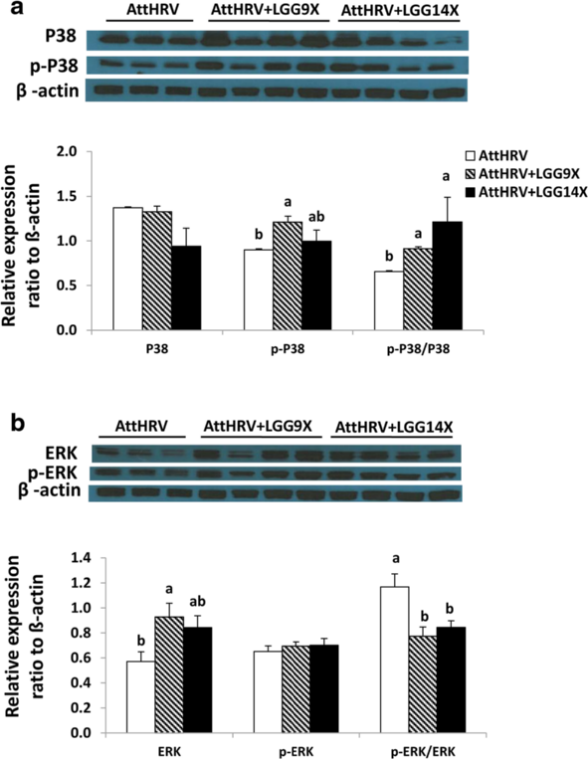
Effect of 9 (AttHRV + LGG9X) or 14 (AttHRV + LGG14X) doses of LGG on levels of innate immune molecules P38 and p-P38 (**a**) and ERK and p-ERK (**b**) in ileal MNCs of the HGM-transplanted Gn pigs. See Fig. 4 legend for statistical analysis

**Fig. 6:**
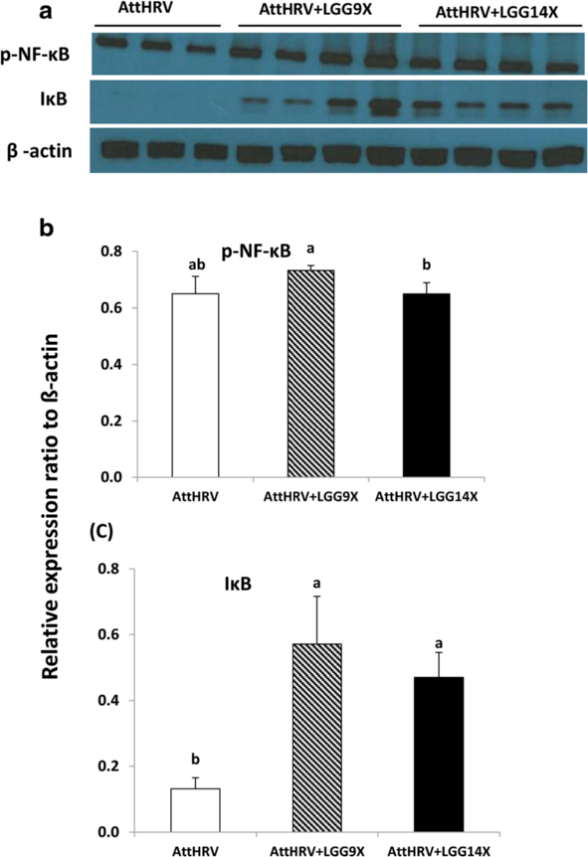
Effect of 9 (AttHRV + LGG9X) or 14 (AttHRV + LGG14X) doses of LGG on levels of immune signaling molecules p-NF-kB and IkBα in ileal MNC of the HGM-transplanted Gn pigs. **a** Western blot image. **b**, **c** Relative expression levels. See Fig. 4 legend for statistical analysis

Signal pathway molecular protein expression in the ileal MNCs were detected by western-blot and presented in Figs. 5 and 6. The relative levels of p-p38 were significantly higher in AttHRV + LGG9X pigs than that in AttHRV pigs. There is no significant difference in p38 among different treatment groups. The AttHRV + LGG9X and AttHRV + LGG14X pigs had significantly higher ratios of p-p38/p38 than the AttHRV pigs; however, there was no significant difference between AttHRV + LGG9X and AttHRV + LGG14X pigs (Fig. 5a).

The levels of ERK were significantly higher in AttHRV + LGG9X pigs than in AttHRV pigs. No significant difference was found in p-ERK among different treatment groups. The ratios of p-ERK/ERK were significantly lower in AttHRV + LGG9X and AttHRV + LGG14X pigs than AttHRV pigs; however, there was no significant difference between AttHRV + LGG9X and AttHRV + LGG14X pigs (Fig. 5b).

The level of p-NF-kB in AttHRV pigs did not differ significantly from that in AttHRV + LGG9X and AttHRV + LGG14X pigs (Fig. 6b). The levels of p-NF-kB were significantly higher in AttHRV + LGG9X pigs than in AttHRV + LGG14X pigs. IkBα were almost undetectable in AttHRV pigs (Fig. 6a). AttHRV + LGG9X and AttHRV + LGG14X pigs had significantly higher IkBα levels than AttHRV pigs; no difference was found between the AttHRV + LGG9X and AttHRV + LGG14X pigs (Fig. 6c).

### Immunohistochemistry for CD80, IFN-γ, p38, p-p38, ERK and pERK in ileal tissue

**Fig. 7:**
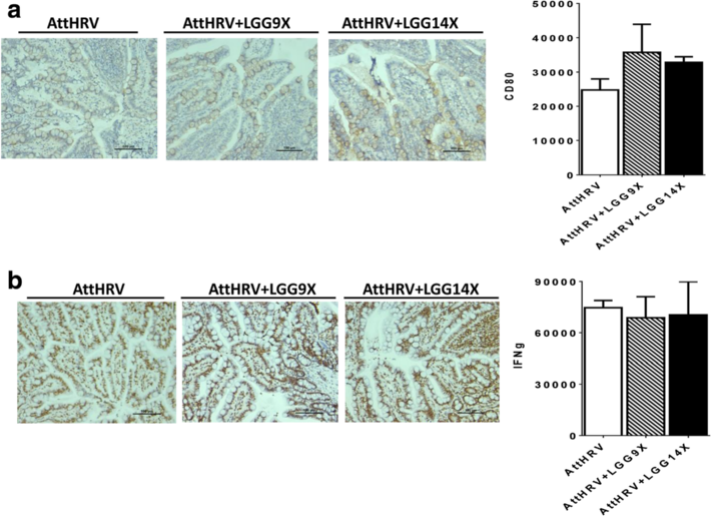
LGG treatment did not alter CD80 and IFN-γ expression in ileal tissues of the HGM-transplanted Gn pigs at PID 28. The levels of CD80 (**a**) and IFN-γ (**b**) were evaluated using semi-quantitative histopathology image analysis (ImageJ) following immunohistochemistry staining of paraffin embedded ileum sections. AttHRV, *n* = 3; AttHRV + LGG9X, *n* = 4; and AttHRV + LGG14X, *n* = 4. See Fig. 4 legend for statistical analysis

**Fig. 8:**
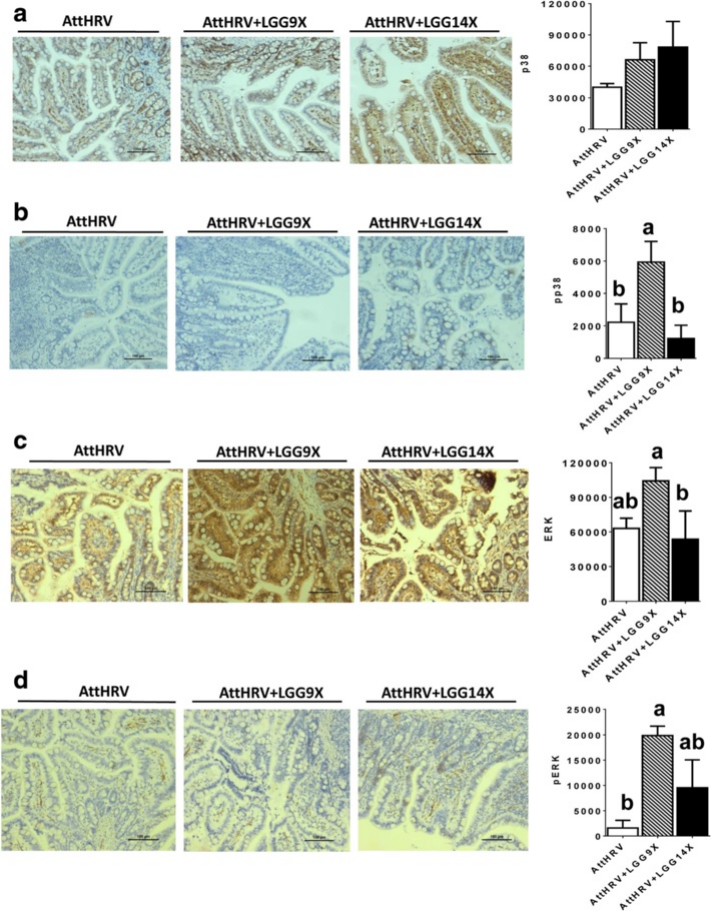
LGG treatment modulates specific MAPK family members in ileal tissues of the HGM-transplanted Gn pigs. The levels of p38 (**a**), pp38 (**b**), ERK (**c**), and pERK (**d**) were evaluated using semi-quantitative histopathology image analysis (ImageJ) following immunohistochemistry staining of paraffin embedded ileum sections. AttHRV, *n* = 3; AttHRV + LGG9X, *n* = 4; and AttHRV + LGG14X, *n* = 4. See Fig. 4 legend for statistical analysis

There is no significant difference in CD80 (Fig. 7a) or IFN-γ (Fig. 7b) expression levels among different treatment groups in immunohistochemistry. Although there is no significant difference in p38 observed among different treatments in immunohistochemistry (Fig. 8a), the AttHRV + LGG9X pigs had significantly higher p-p38 expression levels than the AttHRV and AttHRV + LGG14X pigs (Fig. 8b). The ERK expression levels of the AttHRV + LGG9X pigs were higher than the AttHRV pigs and significantly higher than the AttHRV + LGG14X pigs (Fig. 8c). The AttHRV + LGG9X pigs had significantly higher pERK expression level than the AttHRV pigs (Fig. 8d).

## Discussion

Intestinal microbiota play critical roles in the development of host immune responses [1]. The composition of gut microbes differentially affects the host intestinal mucosal immunity [3]. High levels of variation are apparent in the microbiota of neonates over time and between individuals [14]. Studies using Gn animals have the advantages of highly controlled repeatable experiment design, which reduces inter-individual variation [15]. In addition, Gn pigs with a humanized microbiota better mimic the human infants than the germ-free pigs without gut microbiota. As reported in our previous publications [16, 17], HGM from a single healthy newborn infant was successfully transplanted into newborn Gn pigs. In this study, we confirmed that the bacterial communities in Gn pigs showed high similarity to the human donor in DGGE band patterns. Previous research indicated that human flora associated pigs yielded TGGE (temperature gradient gel electrophoresis) patterns similar to each other as well as to the human donor, but remarkably different from conventionally raised pigs [18].

The relatively high titers of LGG found in feces and LIC of pigs without LGG feeding indicate that there were native LGG in the human donor stool. LGG are often present in intestinal tract of humans [19, 20]. The titers of LGG in LIC were significantly higher in the AttHRV + LGG14x pigs than that in the AttHRV and AttHRV + LGG9X pigs at PPD 28 (PID 23), thus the high dose LGG feeding regimen increased the LGG fecal recovery. The significantly higher LGG titers concurred with significantly enhanced ileal HRV-specific IFN-γ producing T cell responses to the AttHRV vaccine compared to AttHRV and AttHRV + LGG9X pigs reported in our previous publication [16], suggesting the pro-Th1 adjuvant effect of LGG.

Probiotics are recognized to benefit the host through improvement of the balance of intestinal microbiota and through augmentation of host defense system [21, 22]. Probiotics can modulate the intestinal immune system by either directly affecting immune cell activities or through the positive manipulation of the gut microbiota [23]. A clinical study tested the impact of probiotics on the microbiome structure in 6-month old infants fed 1 × 10^9^ CFU/day of LGG and found that communities containing high LGG levels clustered and were associated with a distinct bacterial community composition [24]. In the current study, 14 doses of LGG feeding influenced the structure of the transplanted microbiota whereas 9 doses of LGG slightly increased its richness and diversity. Interestingly, the slightly increased richness and diversity of microbiota in the AttHRV + LGG9X pigs, but not the significantly increased LGG fecal recovery in the AttHRV + LGG14X pigs were associated with the enhanced cytokine and TLR mRNA levels and signaling pathway activation in the ileal MNCs of the HGM Gn pigs. The difference in the two LGG feeding regimens for their timing in relation to AttHRV vaccine inoculation and euthanasia of the pigs may also contribute to the difference besides the LGG dosage. Further studies are needed to evaluate the effect of timing/frequency of probiotic intakes on modulating gut microbiome and immune responses.

Many in vitro studies showed that probiotic bacteria stimulate innate immune cells (i.e., dendritic cells, macrophages) to promote expression of various pro- and antinflammatory cytokines and TLRs [9, 22], but evidence for this stimulatory effect in vivo, especially in the small intestine is limited. In this study, we found significantly higher mRNA levels of IL-6, TNF-α, and IL-10 in the ileal MNCs of the 9 doses, but not 14 doses LGG fed pigs compared with the non-LGG fed pigs. Our results concur with another dose response study reporting that *Lactobacillus rhamnosus* ATCC 7469 feeding for one week at 1 × 10^9^ CFU/dose, but not 1 ×10^14^ CFU/dose upregulated mRNA levels of jejunal IL-2, ileal TGF-β1 and ileal IL-10 after F4 + ETEC challenge in piglets [25].

Toll-like receptors initiate NF-kB and MAPK cascades, which are the defense-related transcriptional factors. Their activation leads to the production of cytokines [26]. Excessive immune responses in the intestinal epithelium can be regulated via multiple mechanisms, including modulations of various TLRs expression and localization, or mediation of downstream immune-related cell signaling activation like NF-kB pathway [27]. These mechanisms are exerted synergistically to maintain immune responses homeostasis in GI tract [27].

The role of TLR2 in the induction of innate responses by probiotic lactobacilli including *L. rhamnosus* in immune cells has been extensively demonstrated. TLR2 recognizes gram-positive bacterial lipoteichoic acid, peptidoglycan and lipoproteins. A previous study showed that LGG enhanced TLR2 mRNA level, and TLR2 was required for NF-kB activation in macrophages [28]. Another recent study confirmed the involvement of TLR2 signaling but not TLR9 in the upregulation of IL-1β, IL-6, IFN-γ and IL-10 mRNA levels induced by *L. rhamnosus* in porcine intestinal antigen presenting cells challenged with virus dsRNA analogue poly (I:C) [29]. In the present study, no significant difference was found in the level of TLR2 mRNA level among treatment groups. TLR9 recognizes bacterial CpG DNA and synthetic unmethylated CpG oligonucleotide mimics (CpG-ODN) [30]. It is known that the genomes of many lactobacilli strains are rich in CpG islands [31]; therefore, lactobacilli may exert a stimulating effect via activation of TLR9 on immune cells. Expression of TLR2, TLR9, and NOD1 mRNA is upregulated in the intestines of pigs pretreated with a low, but not a high, dose of *L. rhamnosus* [32]. In the present study, TLR9 mRNA level was significantly increased in AttHRV + LGG9X pigs compared to the other two groups. The significantly higher TLR9 mRNA level was associated with the significantly higher IL-6, IL-10, and TNF-α mRNA expression in the AttHRV + LGG9X pigs compared to the other groups, indicating that CpG induced TLR9 signaling is likely one of the pathways that LGG stimulated the secretion of the cytokines. Our results are consistent with the observation that *L. rhamnosus* induced cytokine responses (IL-6, IFN-γ, TNF-α, and IL-10) in a TLR9-dependent manner in human blood MNCs, but the role of TLR2 could not be demonstrated [33].

TLR4 recognizes lipopolysaccharide from gram-negative bacteria. In this study, the level of TLR4 mRNA in AttHRV + LGG9X pigs was slightly but statistically higher than that in the non-LGG fed AttHRV pigs. The effect of LGG on TLR4 mRNA levels can only be indirectly since LGG does not contain TLR4 ligands. In general, TLR4 level is downregulated by anti-inflammatory probiotics [34]. In the present study, the increase in TLR4 mRNA levels is consistent with the increased pro-inflammatory cytokine responses in the AttHRV + LGG9X pigs. The possible reasons for the upregulated TLR4 can be the increased richness and diversity of microbiota in the AttHRV + LGG9X pigs and that certain bacteria species in the microbiota promoted TLR4 expression.

In response to inflammatory signals, the MAPK cascade is activated through phosphorylation of p38, ERK, and JNK, which is associated with the activation and translocation of NF-kB from the cytoplasm to the nucleus [35]. NF-kB is known to play a central role in inflammatory responses and is involved in transcriptional regulation of many cytokine genes, including TNF-α [36]. Previous research suggested that both NF-kB and p38 MAPK signaling pathways were important for the production of cytokines and chemokines induced by *L. acidophilus* NCFM [37]. Inhibition of MAPKs family pathway, such as ERK, p38, and JNK, alleviates the production of pro-inflammatory cytokines [38]. Previous research also indicated that p38 MAPK and ERK-1/2 cross-regulate each other such that inhibition of one enhances activation of the other and the effector functions induced in response to different stimuli [39]. We therefore examined whether LGG could induce cytokine responses by activation of p38 MAPK and ERK1/2. In western blot analysis, the AttHRV + LGG9X and AttHRV + LGG14X pigs had significantly higher ratio of p-p38/p38, whereas lower p-ERK/ERK than the AttHRV pigs. This result verified their reciprocal association. The IHC analysis generally verified the result that AttHRV + LGG9X had the highest p-p38 and ERK1/2 in western blot. There was one exception for pERK that the AttHRV + LGG9X pigs had significantly higher pERK than the AttHRV pigs in IHC (Fig. 8d) but not in western blot (Fig. 5b) analysis.

ERK pathway plays key regulatory functions in a diverse spectrum of biological processes such as cell proliferation, differentiation, survival, and motility [40] and has important immunoregulatory role in maintaining homeostasis in the intestine [41, 42]. In this study, 9 doses LGG increased levels of ERK and pERK as observed in western blotting and IHC, respectively, indicating that the activation of ERK pathway by LGG could have protective role against viral infection-induced mucosal injury.

AttHRV + LGG9X pigs had significantly higher levels of p-NF-kB than the AttHRV + LGG14X pigs; but neither pig group significantly differed from the AttHRV pigs. The result indicates that 9 doses LGG further activated the NF-kB, but 14 doses prevented the further NF-kB activation. NF-kB is located in the cytoplasm as an inactive complex bound to IkBα, which is phosphorylated and subsequently degraded, and the degradation of IkBα results in the dissociation of activated NF-kB from IkBα [43]. Pre-treatment of HT29 and T84 polarized cell monolayers using purified DNA from LGG delayed NF-kB activation, stabilized levels of IkBα, and attenuated IL-8 secretion in response to stimulation by Salmonella DNA or TNF-a [44]. In the present study, both LGG dosing regimens significantly increased the IkBα level, which indicates that LGG may have inhibited inflammation by increasing the IkBα level to balance the activation of NF-kB [35]. Therefore, the reduced transcription of TNF-α, IL-6 and IL-10 in the AttHRV + LGG14X pigs may not be due to the lack of impact of LGG on the signaling pathways, rather it reflected the active effect of the higher dose LGG in attenuation of NF-kB activation.

Adequate pro-inflammatory cytokine responses contribute to clearance of pathogen, but excessive inflammatory immune responses lead to tissue injuries. Therefore, an appropriate balance between pro-inflammatory and anti-inflammatory mediators is crucial for an effective and safe response against infection [45]. IL-10 is a potent immunoregulatory cytokine that might be beneficial in the course of infection by attenuating the excessive host inflammatory response induced by upregulated pro-inflammatory cytokines and thus controlling immunopathology. Several studies have demonstrated that induction of the regulatory IL-10 by probiotic lactobacilli such as *L. rhamnosus* plays an important role in controlling inflammatory process upon a viral infection to minimize tissue injury [46]. In the present study, we showed that 9 doses LGG induced higher mRNA level of both pro-inflammatory cytokines (TNF-α, IL-6) and anti-inflammatory cytokine IL-10 when compared with AttHRV and AttHRV + LGG14X. Therefore, the improved production of IL-10 induced by appropriate dose of LGG would allow an efficient regulation of the inflammatory response and avoid tissue damage during intestinal viral infections. Modulation of the NF-kB and p38 MAPK signaling pathways may also have a significant impact on AttHRV vaccine immunogenicity and efficacy via upregulating cytokine productions. Indeed, the increased IL-6 and TNF-α mRNA levels were associated with significantly enhanced intestinal IgA responses in the AttHRV + LGG9X pigs postchallenge as we reported previously [16]. Although the vaccine-induced protection over all did not differ significantly among AttHRV only, AttHRV + LGG9X and AttHRV + LGG14X groups, the AttHRV + LGG9X pigs had the shortest mean duration of diarrhea and virus shedding and significantly lower cumulative fecal diarrhea scores [16].

There is an apparent discrepancy between the significantly increased HRV-specific IFN-γ producing T cell responses in the 14 doses, but not 9 doses, LGG fed HGM pigs [16] and the significantly stronger cytokine, TLR4, TLR9 and p38 MAPK signaling pathway responses in the 9 doses, but not 14 doses, LGG fed HGM pigs. It is important to note the difference in the cell populations studied for the T cell responses (CD3 + CD4+ and CD3 + CD8+ T cells) versus the signaling pathway responses (total MNC and whole ileum tissues). It might have made interpreting the results and discrepancy easier if our studies were performed using sorted T cells and dendritic cells. An in vitro study using human dendritic cells showed that LGG significantly down-regulated p38 expression and negatively regulated NF-kB through down-regulatory effect on miR-146a expression [47]. Although the role of NF-kB pathway in T cell development and function has been well studied [48], the relationship between p38 MAPK signaling pathway and T cell development is not so clear. Further studies are needed to explain the observed discrepancy.

## Conclusions

In conclusion, the two different LGG doses exerted divergent effects on gut microbiota structure and on intestinal immune responses. These results are important since they revealed that the relationship between modulation of gut microbiota and regulation of host immunity using probiotics is complex. More in vivo studies are needed to better understand mechanisms of action of this probiotic strain in its applications as treatment of intestinal infectious or inflammatory disease and as a vaccine adjuvant. An improved understanding of the molecular mechanisms of immunomodulation will facilitate the development of next-generation probiotics and will enhance our understanding of host-microbial interactions.

## Methods

### Attenuated HRV vaccine and LGG

The cell-culture adapted AttHRV Wa strain (G1P1A [8]) was used as the vaccine at a dose of 5 × 10^7^ fluorescent focus forming units (FFU) [49]. The virus titer was determined by using cell culture immunofluorescence (CCIF) assay and was expressed as FFU/ml as described previously [50]. Probiotic LGG (ATCC# 53103) was propagated in lactobacilli MRS broth (Weber, Hamilton, NJ, USA). LGG inoculums were prepared and titrated as we previously described [51].

### Experimental design

Pigs were derived by surgery from near-term sows (Large White cross bred) and maintained in germfree isolator units as described [52]. All pigs were orally inoculated at 1, 2 and 3 day of age with 1 ml of 5 % human stool suspension (a pool collected from a healthy infant at 17–23 days of age) as we described previously [16]. The HGM Gn pigs (both males and females) were randomly assigned to three LGG treatment groups with four pigs in each group as follows: no LGG (AttHRV), 9 doses LGG (AttHRV + LGG9X), and 14 doses LGG (AttHRV + LGG14X). Daily LGG feeding started at 3 days of age for 9 days (3–11 days of age) from 10^3^ to 10^6^ colony forming units (CFU)/dose or for 14 days (3–16 days of age) from 10^3^ to 10^9^ CFU/dose in AttHRV + LGG9X and AttHRV + LGG14X groups, respectively, with 10-fold incremental LGG dose increase every day. Non-LGG fed pigs were given 3 ml of 0.1 % peptone water as we described previously [53].

All pigs were orally inoculated with two doses of the AttHRV vaccine at 5 and 15 days of age. At post AttHRV inoculation day (PID) 28 which is post-partum day [PPD] 33, pigs from each group was euthanized to collect intestinal contents and intestinal tissue for immunohistochemistry (IHC) and for the isolation of mononuclear cells (MNC) from the distal portion of the small intestine (ileum) to study the immune pathway responses.

### LGG counting by real-time PCR

Rectal swabs were collected at PPD10 (PID 5), 15 (PID 10) and 28 (PID 23) and large intestinal content (LIC) were collected at PPD 33 (PID 28) for detection of LGG shedding in the HGM pigs. The fecal samples were treated according to the method by [54].

The strain-specific primers for LGG 16S rDNA are listed in Additional file 1: Table S1. The PCR reaction solution (20 ul) contains 10 ul 2 × SYBY, 0.5 ul 10 pmol/ml Primer (F) and 10 pmol/ml Promer (R), 2ul DNA template, 7 ul H_2_O. The real-time PCR condition is 94 °C 5 min, with a further 40 cycles at 94 °C 15 s, 60 °C 15 s, 72 °C 30s. Fluorescence was measured at start of 72 °C. The pure LGG culture with a known titer was used to prepare standard curve.

### PCR-DGGE analysis

Genomic DNA was obtained as described above. Primer U968-GC and L140lr [55] were used to amplify V6-V8 regions of 16S rDNA. PCR-DGGE was performed and analyzed as we previously described [56]. The Shannon index, H’ of general diversity was calculated according to a previous described method [57] as a parameter for the structural diversity of the microbial community.

### Isolation of MNCs from Ileum

Ileum from all pigs were collected on the day of euthanasia and processed for isolation of MNC as previously described [49]. MNCs for quantitative RT-PCR (cytokine and Toll-like receptors expression) and Western-blot (signal pathway molecular) were subjected to the assays immediately after the isolation of the cells in the same day.

### RNA isolation and real-time RT-PCR for IL-6, IL-8, IL-10, TNF-α, TLR2, TLR4 and TLR9

Total RNA was extracted from the MNC cells (2 × 10^7^) using Trizol LS Reagent (no. 10296–028, Invitrogen) according to manufacturer’s protocols. cDNA was obtained using an Tetro cDNA Systhesis Kit (no. 65043, Bioline USA Inc.). The primers for quantification are shown in Additional file 1: Table S1. The real-time quantitative RT-PCR was done using Sensimix SYBR & Fluorescein Kit (no. QT615-05, Bioline USA Inc.) in a final volume 20 ul, which contained 10 ul 2 × SYBY mix, 1ul RT mix, 0.5 ul 10 pmol/ml of each primer for detection of IL-6, IL-8, IL-10, TNF-α, TLR2, TLR4, TLR9, and β-actin which was used as a housekeeping gene. All PCR reactions were done in duplicated on an iQ5 thermocycler (Bio-Rad). The relative levels of different transcripts were calculated using the ΔΔCt method, and results were normalized based on the expression of β-actin within the same experimental setting.

### Western-blot for detection of signal pathway molecular

The MNC (2 × 10^7^) were resuspended in cell lysis buffer (NP40 Cell Lysis Buffer, Invitrogen) and subjected to SDS-PAGE (Invitrogen). The protein bands were transferred to a nitrocellulose membrane using iBlot® Transfer Stack (Invitrogen) and subjected to immunoblot analysis with the antibodies. Anti-p38 (no. 8690), p-p38 (no. 4511), ERK (no. 4695), p-ERK (no. 4370), p-NF-kB p65 (no. 3033), IkBα (no. 4814), β-actin (no. 4970) antibodies were obtained from Cell Signaling Technology (Danvers, MA, USA). The alkaline phosphatase (AP) linked anti-rabbit IgG (no. 550321B), anti-mouse IgG (no. 550321A), AP chemiluminescent substrate (no. 1208012) and its enhancer were purchased from Invitrogen.

### Immunohistochemistry for detection of signaling pathway molecules in ileal tissue

According to previous methods [58], ileum tissue from Gn pigs were fixed in buffered formalin, embedded in paraffin, and cut into serial sections (4 μm). Deparaffinized and rehydrated sections were boiled in 10 mM sodium citrate buffer (pH = 6.0) for 10 min. After washing twice with Tris-buffered saline with Tween-20 (TBST), sections were blocked with 10 % normal goat serum in TBST for 1 h at room temperature. Sections were incubated with primary IFN-γ (1:300 v/v, Cell Sciences, Canton, MA, USA), CD80 (1:200 v/v, Ancell, Bayport, MN, USA), p38, p-p38, ERK or p-ERK antibodies (1:300 v/v, Cell Signaling Technology, Danvers, MA, USA) overnight at 4 °C. After washing three times with TBST, the HRP-conjugated secondary antibody (Jackson ImmunoResearch, West Grove, PA, USA) was added and the sections were incubated for 1 h at room temperature. The Diaminobenzidine-HRP detection system was added and sections were incubated at room temperature. All incubation steps were conducted in a humidified chamber. Sections were then counterstained with hematoxylin, dehydrated, and cover-slipped. Assessment of positivity of IHC staining [59] were conducted under a microscope (ECLIPSE Ti, Nikon Corp., Tokyo, Japan).

### Statistical analysis

Kruskal-Wallis rank sum test was performed to compare all data, except for the LGG titers, which were compared among treatment groups and time points using Analysis of Variance (ANOVA-GLM) on log10 transferred titers. Statistical significance was assessed at *p* < 0.05. All statistical analysis was performed using SAS program 9.2 (SAS Institute, INC, USA).

## Abbreviations

AttHRV, attenuated human rotavirus; CFU, colony forming units; DGGE, Denaturing gradient gel electrophoresis; ERK, extracellular signal-regulated kinase; Gn, Gnotobiotic pigs; HGM, human gut microbiota; LGG, *Lactobacillus rhamnosus* GG; MAPK, mitogen-activated protein kinase; NF-kB, nuclear factor kB; TLR, Toll-like receptors

## Additional file

Additional file 1: Table S1. The primers used in this study. (PDF 54 kb)
