# Supplementary material for: Lactobacillus rhamnosus GG modulates innate signaling pathway and cytokine responses to rotavirus vaccine in intestinal mononuclear cells of gnotobiotic pigs transplanted with human gut microbiota
Source: BMC Microbiol. 2016 Jun 14;16:109. doi: 10.1186/s12866-016-0727-2 (PMC4908676; doi:10.1186/s12866-016-0727-2)
Supplement: Additional file 1: Table S1. — The primers used in this study. (PDF 54 kb) [file 12866_2016_727_MOESM1_ESM.pdf]

Additional file 1: Table S1. Primers used in this study

Primer for	Forward sequence	Reverse sequence	Reference
LGG	5′-CGCCCTTAACAGCAGTCTTCAAAT-3′	5′-ACGCGCCCTCCGTATGCTTAAACC-3′	[1]
IL6	5′-TGGATAAGCTGCAGTCACAG-3′	5′-ATTATCCGAATGGCCCTCAG-3′	[2]
IL8	5′-GCTCTCTGTGAGGCTGCAGTT-3′	5′-TTTATGCACTGGCATCGAAGTT-3′	[2]
IL10	5′-TGGGTTGCCAAGCCTTGT-3′	5′-GCCTTCGGC ATTACGTCTTC-3′	[2]
TNF-α	5′-CGACTCAGTGCCGAGATCAA-3′	5′-CCTGCCCAGATTCAGCAAAG-3	[2]
TLR2	5′-ACATGAAGATGATGTGGGCC-3′	5′-TAGGAGTCCTGCTCACTGTA-3′	[2]
TLR4	5′-CTCTGCCTTCACTACAGAGA-3′	5′-CTGAGTCGTCTCCAGAAGAT-3′	[2]
TLR9	5′-GTGGAACTGTTTTGGCATC-3′	5′-CACAGCACTCTGAGCTTTGT-3′	[2]
β-actin	5′- CATCACCATCGGCAACGA-3′	5′- GCGTAGAGGTCCTTCCTGATGT-3′	[2]

[1] Ahlroos T, Tynkkynen S: **Quantitative strain-specific detection of *Lactobacillus rhamnosus* GG in human faecal samples by real-time PCR**. *Journal of applied microbiology* 2009, **106**(2):506-514.

[2] Moue M, Tohno M, Shimazu T, Kido T, Aso H, Saito T, Kitazawa H: **Toll-like receptor 4 and cytokine expression involved in functional immune response in an originally established porcine intestinal epitheliocyte cell line**. *Biochim Biophys Acta* 2008, **1780**(2):134-144.
